# Natural and artificial selection of multiple alleles revealed through genomic analyses

**DOI:** 10.3389/fgene.2023.1320652

**Published:** 2024-01-08

**Authors:** Jana Biová, Ivana Kaňovská, Yen On Chan, Manish Sridhar Immadi, Trupti Joshi, Kristin Bilyeu, Mária Škrabišová

**Affiliations:** ^1^ Department of Biochemistry, Faculty of Science, Palacký University in Olomouc, Olomouc, Czechia; ^2^ MU Institute for Data Science and Informatics, University of Missouri-Columbia, Columbia, MO, United States; ^3^ Christopher S. Bond Life Sciences Center, University of Missouri-Columbia, Columbia, MO, United States; ^4^ Department of Electrical Engineering and Computer Science, University of Missouri-Columbia, Columbia, MO, United States; ^5^ Department of Biomedical Informatics, Biostatistics and Medical Epidemiology, University of Missouri-Columbia, Columbia, MO, United States; ^6^ United States Department of Agriculture-Agricultural Research Service, Plant Genetics Research Unit, Columbia, MO, United States

**Keywords:** genetic variation, GWAS, causal gene, causative mutation, alleles, breeding, soybean

## Abstract

Genome-to-phenome research in agriculture aims to improve crops through *in silico* predictions. Genome-wide association study (GWAS) is potent in identifying genomic loci that underlie important traits. As a statistical method, increasing the sample quantity, data quality, or diversity of the GWAS dataset positively impacts GWAS power. For more precise breeding, concrete candidate genes with exact functional variants must be discovered. Many post-GWAS methods have been developed to narrow down the associated genomic regions and, ideally, to predict candidate genes and causative mutations (CMs). Historical natural selection and breeding-related artificial selection both act to change the frequencies of different alleles of genes that control phenotypes. With higher diversity and more extensive GWAS datasets, there is an increased chance of multiple alleles with independent CMs in a single causal gene. This can be caused by the presence of samples from geographically isolated regions that arose during natural or artificial selection. This simple fact is a complicating factor in GWAS-driven discoveries. Currently, none of the existing association methods address this issue and need to identify multiple alleles and, more specifically, the actual CMs. Therefore, we developed a tool that computes a score for a combination of variant positions in a single candidate gene and, based on the highest score, identifies the best number and combination of CMs. The tool is publicly available as a Python package on GitHub, and we further created a web-based Multiple Alleles discovery (MADis) tool that supports soybean and is hosted in SoyKB (https://soykb.org/SoybeanMADisTool/). We tested and validated the algorithm and presented the utilization of MADis in a pod pigmentation *L1* gene case study with multiple CMs from natural or artificial selection. Finally, we identified a candidate gene for the pod color *L2* locus and predicted the existence of multiple alleles that potentially cause loss of pod pigmentation. In this work, we show how a genomic analysis can be employed to explore the natural and artificial selection of multiple alleles and, thus, improve and accelerate crop breeding in agriculture.

## 1 Introduction

**FIGURE 1:**
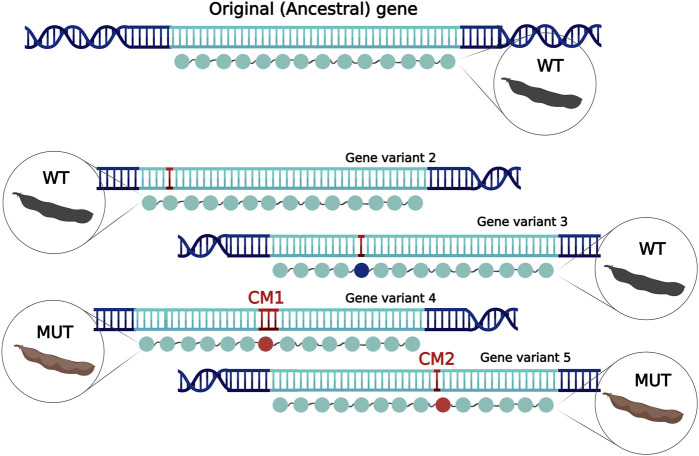
Simplified scheme representing the isolated emergence of multiple alleles as a part of evolution during natural selection or domestication. The original protein coding sequence of the ancestral gene may accumulate many mutations (red in the DNA sequence) that can be synonymous (no color change in the protein sequence) and/or non-synonymous (dark blue in the protein sequence) that do not impact protein function, whereas other non-synonymous mutations may be modifying or disrupting (red in the protein sequence). Such alleles can be beneficial and, therefore, undergo natural selection or artificial selection. This example shows the emergence of two geographically or temporally isolated CMs (CM1—a small insertion; CM2—a SNP) that can lead to disturbed pigmentation of pods.

Natural and artificial selection guides crop breeding and improvement. Therefore, a key determinant is a thorough understanding of crop diversification through evolution and domestication from wild relatives and early improved types (landraces). Next-generation sequencing boosted the methods for the exploration of genetic diversity and, thus, contributed to our better understanding of convergent evolution in crops, as reviewed by Pickersgill (2018). One of the methods that has been used for the exploration of diversification and for identification of loci associated with a phenotype is quantitative trait locus (QTL) mapping. This method is, however, limited by the creation and use of bi-parental populations and, therefore, rarely leads to the precise identification of causal genes that underlie a studied phenotype. Lately, genome-wide association study (GWAS) has been a driving force of new gene discoveries. With improved genotypic data size and quality, GWAS has successfully identified genes important for the initial evolutionary diversification of cultivated crop species (reviewed by Meyer and Purugganan (2013)). By definition, convergent evolution can mean two things: multiple genes that control a single trait, where variants in these isolated genes underlie the same phenotype or a single gene that controls the trait, where multiple independent CMs underlie a phenotypic change. In this work, we focus on a single gene with multiple CMs, the same nature as in the case of convergent evolution of the well-known example from human genetics, lactase persistence (Tishkoff et al., 2007), and its ethnically specific, geographically isolated emergence of adult-functioning alleles. In crops, convergent evolution with subsequent selection has played a crucial role in the domestication of ancestral varieties and improvement of landraces to elite cultivars. Independent alleles of a single gene can impact the resulting phenotype differently. Multiple alleles of a gene can either underlie two discrete categories of phenotypes (wild type; WT, the ancestral and predominantly functional phenotype; and mutant; MUT, predominantly non-functional allele) or a spectrum of phenotypes, as found in allelic series. Figure 1 illustrates a simplified scheme of multiple allele emergence in an example of soybean pod wall pigmentation.

In this work, we focus on soybean, where selection has been well-studied and characterized, with selection signals observed in almost all reported domestication-related regions (Zhou et al., 2015). In addition to its global importance as a premier oil and protein crop, soybean has an almost exclusive self-pollinating nature and historically strong geographic-related patterns of distribution, making it an attractive system for investigations connecting genes with phenotypes. Soybean genes have been cloned that confirm many of the identified selection signals, although the bi-parental genetic methods and current association studies used for candidate gene identification lack the ability to test for multiple CMs. In GWAS-driven discoveries, the presence of more than just a single CM complicates candidate gene discovery, even though the existence of multiple CMs is common in crop species due to natural and artificial selection that has led to their convergent evolution. In soybean, multiple CMs have been discovered in many domestication-related genes, such as the stem termination gene *Dt1* (Tian et al., 2010); maturity genes *E1* (Xia et al., 2012), *E3* (Watanabe et al., 2011; Xia et al., 2012), *E4* (Tsubokura et al., 2013), and *E6/J* (Lu et al., 2017; Lu et al., 2020); seed coat color gene *R* (Gillman et al., 2011); pubescence color gene *Td* (Yan et al., 2020); *Tof11* and *Tof12* (Lu et al., 2020); and *Rhg* resistance genes to the soybean cyst nematode (Patil et al., 2019).

In our previous work, in an effort to increase the power of GWAS-driven discoveries, we developed an additional GWAS evaluation criterion named accuracy (Škrabišová et al., 2022). Accuracy serves as a measure of direct correspondence between variant positions and phenotypes with binomial distribution. We utilized our accuracy concept for a selection of known soybean genes with cloned CMs and proved that this approach can successfully identify CM and, thus, assist in decreasing the number of false positive candidate genes (Škrabišová et al., 2022). In GWAS, for a gene with multiple independent CMs, often, only the most frequent CM is indicated by the associated genomic locus, as in the case of the soybean main stem termination gene *Dt1* (Fang et al., 2017). We employed our synthetic phenotype association study (SPAS) (Škrabišová et al., 2022) and proved a direct correspondence between a previously identified *Dt1*-tagging low-density genotype marker (Bandillo et al., 2017) and the most frequent R166W *dt1* allele (Tian et al., 2010) by calculating accuracy. This led us to finding that accuracy values can be near perfection (100%) or that they can be decreased to some extent or close to randomness (approximately 50%). We further utilized SPAS and accuracy to investigate non-causative mutations (prior-founder effect emergence), multiple independent CMs, and tagging low-density genotyping markers for soybean cyst nematode resistance genes (Mahmood et al., 2023), and discovered a pattern that is specific to genes with multiple CMs when the average accuracy is perplexing but nearly perfect for the WT phenotype.

Recently, for soybean, a causal gene was identified for the pod color *L1* locus that causes the loss of black pigments in pod walls (Lyu et al., 2023). This visually distinguishable trait was biochemically characterized in the 1920s (Nagai, 1921; Owen, 1927) and further linked with agronomically and nutritionally important traits such as pod shattering, protein content, and pod borer susceptibility (Batchelor et al., 1997; Zhao et al., 2008; Lyu et al., 2023). Despite the tremendous efforts that resulted in candidate gene selection (He et al., 2015; Li et al., 2016; Torkamaneh et al., 2018; Chang et al., 2021; Lemay et al., 2023), none of the earlier candidates was confirmed to functionally underlie black pod color pigmentation until *Glyma.19G120400* was identified recently (Lyu et al., 2023). After more than 100 years of effort, this gene was cloned. *Glyma.19G120400* codes for eucomic acid synthase, and identifying this gene was a difficult undertaking complicated by the presence of six CMs in the *L1* candidate (Lyu et al., 2023). In soybean, loss of black pigments may result in tan or brown pod coloration (Nagai, 1921; Owen, 1927) depending on the status of another allele contributing to pod color, *L2* (Bernard, 1967). Several candidate genes were proposed for *L2* (Song et al., 2004; Lemay et al., 2023); however, none of them has been confirmed yet.

In this work, we show how a genomic analysis can be employed to explore the natural and artificial selection of multiple alleles. We present the development of an algorithm that computes and scores combinations of variant positions in a single candidate gene with the aim of identifying multiple CMs. Here, we demonstrate the validation of our genomic analysis on an example of a recently cloned gene *L1* with multiple CMs and further propose a new candidate gene for *L2* by identifying multiple CMs.

## 2 Materials and methods

### 2.1 MADis development

The logic of MADis is in scoring the mutative arrangements of a number of variant positions in respect to their direct correspondence to the phenotype where the best score achieved represents the best combination of specific variant positions that explain phenotypes for the highest number of accessions. MADis uses two inputs, a genotype in vcf format and a binary phenotype. MADis uses a binary phenotype of two categories, wild-type (WT) and mutant (MUT), and a binary genotype (reference, REF; alternate, ALT). In MADis, a user can specify which allele of a single position is coded as REF, referred to herein as being the ancestral gene variant (functional in general) from which the other evolutionary younger alleles emerged and that can bear independent CMs and, thus, create various non-functional multiple alleles. The output of MADis permutation is a list of combinations of variant positions sorted descending by score in a tabular format.

The MADis tool was developed in Python 3.8 programming language and utilizes the following Python packages: pandas (1.3.5; https://pandas.pydata.org/), NumPy (2.21.5; https://numpy.org/), and itertools (3.11.4; https://docs.python.org/3/library/itertools.html). The MADis Python package consists of two essential functions that take and analyze the input data. The first function import_vcf(file, gene_nam) enables the import of genotype data and transforms them into the genotype numerical binary matrix. The user-provided phenotype data input format requires a tab-delimited file format with two columns that list accession names and phenotype in numerical values (WT = 0; MUT = 1). After the data input, there are data pre-calculation steps. For genotype data, these include position and sample sorting and transformation of the final genotype data into a binary matrix. The genotype positions are sorted based on the predicted mutation effect on the gene. Only the modifying variant positions with the predicted high impact on the protein level are selected for further analysis. The sample sorting is based on the availability of the phenotype, where only the samples with the valid (WT or MUT) phenotype are included in the analysis and the samples with the missing phenotype values are omitted. The core calculation of the MADis algorithm is the scoring, which is incorporated into the second function *make_score(geno*, *phen*,*N*,*d_score = d_score)* and represented by Eq. 1:

$${score}_{comb}=\sum_{i=1}^{n}{ssc}_{i}.$$ (1)

In Eq. 1, *score*_*comb*_ is a sum of the scores for individual samples and *n* is the number of samples in the analysis for *i* sample order number in the tested combinations *comb*. The individual sample score for a combination of variant positions *ssc* is based on Pascal’s triangle and is calculated as shown in Table 1. Here, we assume that according to our understanding of how multiple independent CMs arise during genome evolution, the probability of the existence of multiple independent CMs in one sample is low. This is incorporated in the individual sample score by assigning a positive score (1) for samples with a WT phenotype with only REF alleles in a tested combination of variant positions and, simultaneously, for samples with a MUT phenotype and exactly one ALT allele that could explain the phenotype transition from WT to MUT. The presence of ALT alleles in an individual sample that differs from the initial assumption is, therefore, a reason for a negative score assignment. The value of the negative score depends on how much an individual sample deviates from our understanding of the multiple CM emergence (in other words, one CM for a sample with the MUT phenotype and no CM present in any of the samples with the WT phenotype).

**TABLE 1:** Individual sample score (*ssc*) matrix for the combination of increasing counts of variant positions. The individual score is calculated for every sample and is determined by the number of all ALT allele genotypes for WT and MUT phenotypes of a sample.

Count of variant positions with the ALT allele genotype in the tested combination for an individual sample	Score of an individual sample with the WT phenotype	Score of an individual sample with the MUT phenotype
0	1	−1
1	−1	1
2	−3	−3
3	−6	−6
4	−10	−10
5	−15	−15
6	−21	−21
7	−28	−28

The theoretical maximal *score*_*comb*_ value is a score that is given by individual sample scores of both phenotypic categories that result in 100% explained phenotypes of all samples in a given dataset. Therefore, the theoretical maximal *score*_*comb*_ value is dataset size-specific and independent of the number of positions in any of the combinations. Thus, the theoretical maximal *score*_*comb*_ value enables the comparison of *score*_*comb*_ of the tested combinations and genes. The MADis results are presented in a table-based format for the selected number of the best-scored combinations. The table includes calculated values for analyzed combinations such as *score*_*comb*_, maximal *score*_*comb*_, and the percentage of explained samples, as well as the count of included WT and MUT samples, and the count of explained WT and MUT samples. The assessment duration for varying combinatorial quantities within our analysis, encompassing a dataset of nearly 700 samples (691) distributed across 16 discrete positions, required less than 5 s to compute scores for the complete spectrum of combinations of two, three, or four and less than 20 s for the combinations of five and six. The tool is publicly available for any species as a Python package on GitHub (https://github.com/Biovja/MADis/) with available demo files and descriptions.

Apart from the Python package, a user-friendly and publicly accessible web-based Soybean MADis Tool powered by the Soy1066 dataset (Chan et al., 2023) has also been created. The architecture of the Soybean MADis Tool includes the MySQL database that links the SoyKB web portals (Joshi et al., 2012; Joshi et al. 2014; Joshi et al. 2017), back-end processing code in PHP, and front-end user interfaces with interactive components and visualizations developed with HTML, CSS, and JavaScript. The purpose of the Soybean MADis Tool is for users to provide genes and phenotypes to perform calculations using the MADis algorithm and visualize the results in interactive visualizations to help researchers select the best explainable variant position combinations and advance their research. The MADis initial round of calculations starts with combinations of two variant positions. This step, when only combinations of two are considered in the first round and, for a higher number of combinations, the second round follows, is specific to the MADis web tool. Splitting the analysis into two steps in the web tool is required because of the web tool’s limitation in computational time and space, unlike the analysis running on the server. The two-step analysis with the second round of the MADis calculation approach is developed for users to select only the important variant positions in the first round results to avoid heavy computational costs. This approach can not only reduce computational time but also reduce resource allocation to increase the computational efficiency. The total data size for computing with combinations of two variant positions can be calculated using the combination formula (Eq. 2) as follows:

Total data size=Cn2, (2)

where *n* denotes the total number of variant positions in a gene. For a second round of MADis calculations, users select the preferred combinations in the initial round of MADis results and perform subsequent calculations for up to the maximum of combinations of seven variant positions. The total data size to compute can be calculated using the following formula (Eq. 3):

Total data size=Cm7 +Cm6 +Cm5 +Cm4 +Cm3 +Cm2, (3)

where *m* is the total number of variant positions selected for computing in this round. The Soybean MADis Tool is presently available on the SoyKB web portal at https://soykb.org/SoybeanMADisTool/.

### 2.2 Genotypes and phenotypes

In this study, we used our Soy1066 dataset (Chan et al., 2023) available at https://soykb.org/public_data.php as a genotype input. This dataset represents diverse soybean accessions with geographical distribution capturing the worldwide soybean population of distinct improvement statuses (*Glycine soja* and *Glycine max*, landraces, elite lines, and North American cultivars). For phenotypes, we pulled pod color status from the publicly available USDA Soybean Germplasm Collection (GRIN, Urbana, IL). From the Soy1066 dataset, there were a total of 732 accessions with available phenotypes, black, brown, or tan pod-colored, which were utilized in the analysis. For the *L1* analysis, to answer the scientific question of whether *L1* underlies the loss of black pigmentation of pod color, the available phenotypes were categorized as WT for black (127) and MUT for brown or tan (605) pod-colored accessions. For the *L2* analysis, to answer the scientific question of whether *L2* underlies the loss of brown pigmentation of pod color in accessions with a non-functional *l1* allele, the used phenotypes were categorized as WT for brown (418) and MUT for tan (187) pod-colored accessions.

### 2.3 MADis validation

For the MADis tool, the different types of scoring tables for the determination of *ssc* were tested. The *ssc* scoring table and MADis validation were performed for both types of soybean-based testing data: artificially created and previously known multiple CM case examples. The first round of validation was performed on artificially created data while modeling several multiple CM scenarios (low-frequency CMs, balance-frequency CMs, high-frequency CMs, and low-frequency CM combinations). For MADis validation using the example cases, the previously known soybean multiple CMs were used. The genotype data from the Soy1066 dataset (Chan et al., 2023) and phenotype data from the USDA Soybean Germplasm Collection (GRIN, Urbana, IL), both publicly available, were used. During the validation, the MADis analysis was performed for the known causative gene, genes in close distance to causative genes, and randomly chosen genes. The chosen ssc scoring table was selected based on the best results in determining the multiple CMs in both types of tested data.

### 2.4 Analysis of alleles

The Allele Catalog Tool (Chan et al., 2023) was used to analyze the allele distribution in *L1* and *L2*. The output table was downloaded and streamlined to display only the positions identified by MADis. Subsequently, the simplified table was reconstructed, and the visualization was created using Biorender.com. For visualization of the distribution of pod-color phenotypes of each of the alleles displayed, we used pod-color phenotypes from GRIN and visualized the information through pie charts using Biorender.com.

### 2.5 Identification of *L1* alleles by MADis

Within the validation process, the previously known case studies for the multiple allele CMs were used. The various example cases (not included in the publication) were tested during validation for MADis to undergo examination against a more heterogeneous array of testing data. The example case used as a validation example in the publication was based on the recently published *L1* (Lyu et al., 2023). The MADis analysis was performed using all variant positions of the candidate gene (*Glyma.19G120400*) that were predicted as the modifying variant positions with the predicted high impact on the protein level (18). For the analysis, only the samples with the available WT or MUT phenotype were used (732 samples from the 1,066 samples included in the Soy1066 dataset). For the *L1* analysis, the available phenotypes were categorized as WT for black (127) and MUT for brown or tan (605) pod-colored accessions. The combination of positions with the highest score in the MADis analysis was used as the analysis result.

### 2.6 Identification of the *L2* causal gene

For *L2* identification, we used the previously published *L2* locus, discovered using SoySNP50K, determined by the marker ss715586818 on chromosome Chr03 at position 536,829 (Wm82.a2.v1) (Bandillo et al., 2017). We analyzed a 2-Mbp region around the ss715586818 marker for specifying and focusing on the previously described *L2* locus in the soybean genome. The analysis was performed by AccuCalc (Biová et al., 2023), with a focus on modifying variant positions. The phenotypes used for L2 identification were categorized as WT for brown (418) and MUT for tan (187) pod-colored accessions. According to the AccuCalc result, we predicted the potential occurrence of the multiple allele CMs for the tested trait and used MADis for the hypothesis verification.

### 2.7 Identification of *L2* alleles by MADis

The MADis analysis for *L2* was performed similar to the *L1* MADis analysis. All variant positions of the candidate gene (*Glyma.03G005700*) that were predicted as the modifying variant positions with the predicted high impact on the protein level (33) were used in the analysis. For the analysis, only the samples with available WT or MUT phenotypes were used (605 samples from the 1,066 samples included in the Soy1066 dataset). For the *L2* analysis, the available phenotypes were categorized as WT for brown (418) and MUT for tan (187) pod-colored accessions. The combination of positions with the highest score in the MADis analysis was used as the analysis result.

### 2.8 Geographic distribution

We obtained region data for accessions with functional and non-functional alleles underlying pod-color phenotypes utilizing the Soybean Allele Catalog Tool with the Soy1066 dataset (Chan et al., 2023). First, the accessions were divided into three groups—functional *L1*, non-functional *l1*, and non-functional *l2—*and their regions of origin were depicted on a world map using an Excel Cartogram Data Generator. For each region, we calculated the number of accessions carrying a certain allele and plotted these data into pie charts to show the geographical distribution of non-functional *l2* alleles. Based on the frequency of accessions with a certain allele, regions with low frequency (two or less) were omitted from the visualization (Supplementary Table S2—geographical information *L2*). A map-based visualization was created using Biorender.com.

## 3 Results

In our prior work, we developed new association strategies with the assistance of accuracy analysis to leverage whole-genome sequence datasets in the quest for gene identification (Škrabišová et al., 2022). In some cases, we observed unusually low accuracy values for the highest associated positions in a candidate gene. A missing feature of our strategies was the ability to test for multiple variant positions that defined independent alleles of the gene. To capture all possible additional alleles that contribute to a desired phenotype change, we developed the MADis tool and tested the tool on previously known multiple CM cases (not shown), presenting its validation on the recently identified *L1* locus with multiple CMs in *Glyma.19G120400* that encodes eucomic acid synthase, the pod wall pigmentation causal gene (Lyu et al., 2023).

### 3.1 MADis tool

**FIGURE 2:**
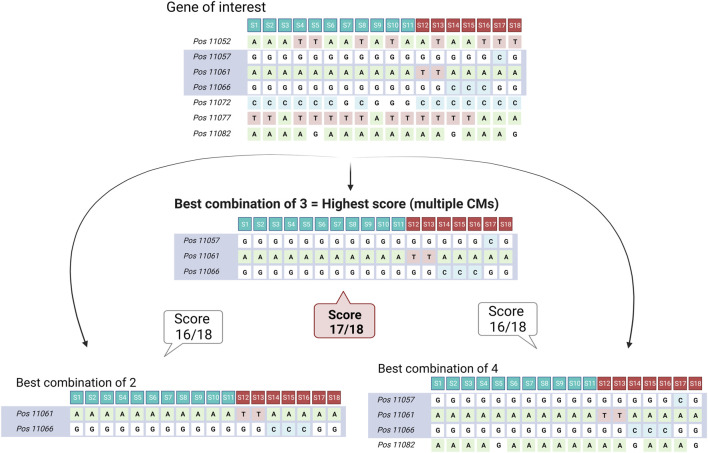
Simplified scheme of the MADis algorithm. The MADis tool tests combinations of variant positions in a gene with the aim of reaching the highest score that would explain most of the phenotypes in the dataset (samples S1–S11 in blue have the WT phenotype, and samples S12–S18 in red have the MUT phenotype). This is performed for default combinations of 2–7 variant positions of all positions present in the gene as a permutation that results in the identification of the best combination of variant positions (gray, potential CMs) that was assigned the highest score. In this simplified scheme, the best combinations of 2–4 variant positions are shown with scores that nearly reached the maximal score for this example (18). In this demonstrative case, the combination of the three specific variant positions in gray reached the highest score that explains the majority of phenotypes, and therefore, this gene would be considered causal with multiple alleles.

The MADis tool concept is based on scoring combinations of variant positions that explain most of the binarized phenotype and selecting the combinations of the highest score that are considered potential multiple CMs (Figure 2). The tool was validated in the analysis with artificially created and previously known multiple CM case examples, thus modeling several multiple CM scenarios (low-frequency CMs, balance-frequency CMs, high-frequency CMs, and low-frequency CM combinations). The tool is *a priori* coding sequence-centered and limited to modifying variants only, which enabled its implementation as a web-based tool on SoyKB.org (Joshi et al., 2012; Joshi et al. 2014; Joshi et al. 2017). This tool is freely accessible through standard web browsers, making it a user-friendly resource. It holds significant value for the soybean research community, offering the capability to conduct data analysis using the MADis algorithm on curated soybean datasets. A detailed description of the Soybean MADis Tool is available at https://github.com/yenon118/SoybeanMADisTool and in the user manual at https://soykb.org/SoybeanMADisTool/. We also developed the MADis Python package version that enables more complicated or time- and space-consuming analysis, e.g., analysis on a promoter region or multiple gene analysis, and this package can be used for private datasets of any species. The MADis package is available on GitHub (https://github.com/Biovja/MADis/) with demo files and descriptions.

### 3.2 *L1* case study for MADis identification of multiple alleles

The *L1* locus responsible for the black pod wall color was recently revealed to be a gene *Glyma.19G120400* encoding a hydroxymethylglutaryl-coenzyme A (CoA) lyase-like (HMGL-like) domain protein, eucomic acid synthase (Lyu et al., 2023). Lyu et al. originally used biparental mapping but ultimately identified seven polymorphisms and four haplotypes (H1–H4), with three haplotypes suggested to play a role in the loss of pod pigmentation. Using the Soybean Allele Catalog Tool (Chan et al., 2023), we identified 18 different alleles in *L1* in Soy1066 (Supplementary Table S3—Allele Catalog Tool Output *L1*), including the seven recently detected polymorphisms (R31C, G40D, T196S, V276A, N418D, K405fs, and L54_A55insRI—functional insertion of arginine and isoleucine, herein referred to as RI_indel) (Lyu et al., 2023).

The recent cloning and extensive list of available alleles and phenotypes made the *L1* gene attractive for a multiple allele case study utilizing the MADis tool. We performed permutation for 732 accessions with a known pod-color phenotype and all modifying variant positions in the *Glyma.19G120400* gene. Based on the scores obtained for the combinations of variant positions (Supplementary Table S1—L1 *Glyma.19g120400* MADis result), we identified RI_indel as the ancestral allele of accessions with an RI insertion, whereas those two amino acids have been deleted in the reference genome Williams 82 (Williams 82.a2.v1). In addition to the two alleles defined by the reference or RI_indel position, we identified five other positions (Table 2) that are part of unique alleles, all of which led to missense or frameshift mutations and also contained the RI_indel variant (R31C, G40D, L381fs, K405fs, and E402fs). These six positions were a perfect phenotype correspondence for more than 98% of the analyzed accessions, achieving the highest score, and therefore, they were selected as the most probable candidates for CMs. These alleles were only found in *G. max*, which suggests that they were artificially selected during the domestication process of soybean.

**TABLE 2:** Top five best-scored MADis outputs for *Glyma.19G120400*. The MADis results for the analysis of potential multiple CMs in *Glyma.19G120400* affecting the pod color trait. In the analysis, the 127 black pod-colored accessions and 605 brown and tan pod-colored accessions were used for MADis prediction. The MADis result was a perfect correspondence for more than 98% of analyzed accessions, implying the discovered multiple CMs in *Glyma.19G120400* as a strong candidate.

Order	Variant positions in *Glyma.19G120400*	Number of variant positions in the combination	Score	Black (*n* = 127) explained	Brown and tan (*n* = 605) explained	Explained phenotype (%)
1	37,806,091	6	704	124	594	98.1
	37,806,119					
	37,806,160					
	37,819,253					
	37,819,382					
	37,819,390					
2	37,806,091	5	702	124	593	98.0
	37,806,119					
	37,806,160					
	37,819,382					
	37,819,390					
3	37,806,091	7	702	123	594	98.0
	37,806,119					
	37,806,145					
	37,806,160					
	37,819,253					
	37,819,382					
	37,819,390					
4	37,806,091	7	702	123	594	98.0
	37,806,119					
	37,806,160					
	37,806,193					
	37,819,253					
	37,819,382					
	37,819,390					
5	37,806,091	5	700	124	592	97.8
	37,806,119					
	37,806,160					
	37,819,253					
	37,819,382					

**FIGURE 3:**
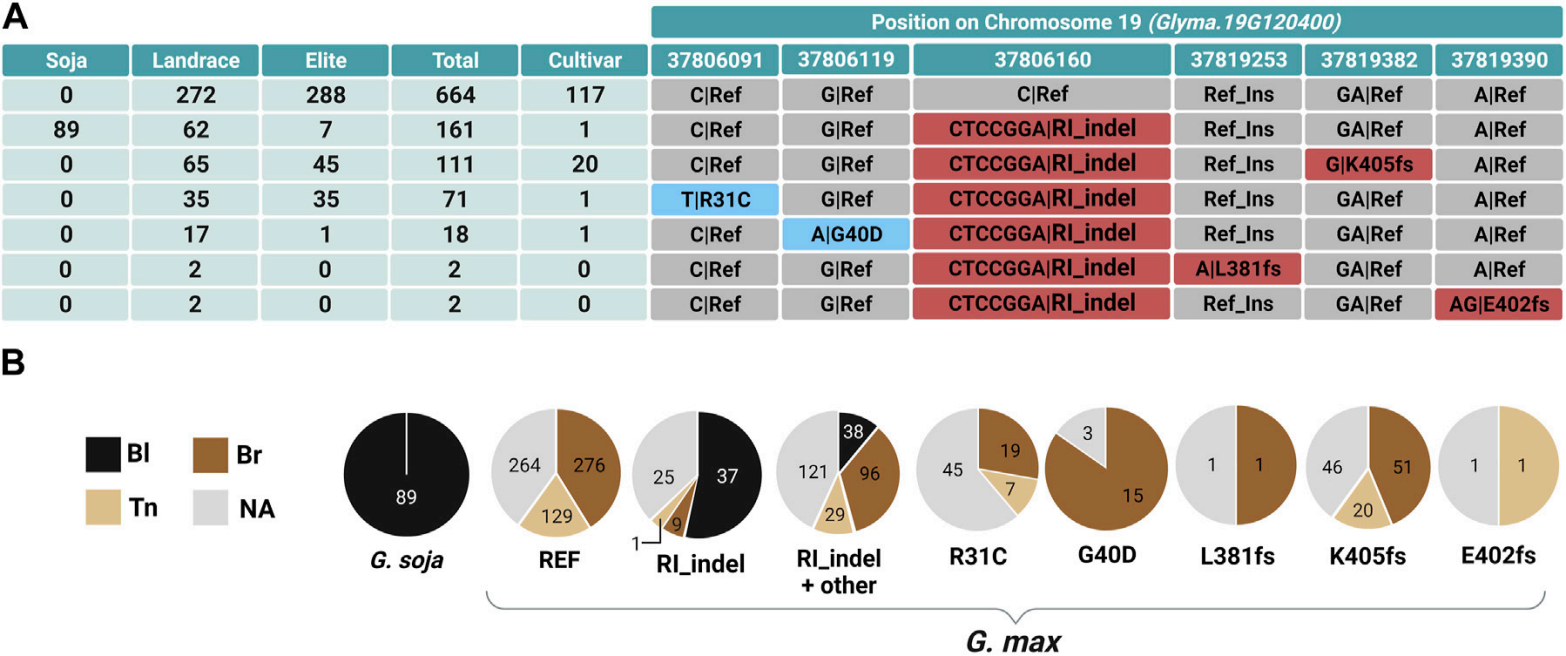
Allele distribution in *Glyma.19G120400* in Soy1066 and its correlation with different pod-colored phenotypes. **(A)** The table shows 161 total accessions with what is presumably the ancestral functional allele (second row) containing only the modifying variant for the RI_indel, as well as six alleles that each contain one of the six CMs identified by MADis. The modifying genomic positions in *Glyma.19G120400* and frequency counts of accessions in different improvement status categories carrying each allele are presented. Positions with SNPs leading to amino acid change are highlighted in blue, while positions with frameshift mutations are indicated in red. Gray designates the reference (REF) allele, based on the Williams 82 reference genome. **(B)** Pie charts represent the distribution of pod-colored phenotypes for *G. soja* and *G. max* accessions with specific alleles.

After the implementation of MADis analysis (Supplementary Table S1—L1 *Glyma.19G120400* MADis result), the 18 detected alleles were subsequently narrowed down based on MADis scoring to a combination of six alternative alleles in the gene that were identified as non-functional (Lyu et al., 2023) and, thus, responsible for the loss of black pod color (Table 2). The six non-functional alleles and the functional ancestral allele, together with the accession counts in Soy1066, are shown in Figure 3A, whereas Figure 3B shows the distribution of pod-color phenotypes for each of the alleles. We observed that an RI insertion (RI_indel) was present in all *G. soja* accessions that are typical for their black pod color. Among 111 *G. soja* accessions in Soy1066, 89 possess exclusively the RI_indel allele. In one *G. soja* accession, a WI_indel occurs in place of the RI_indel (Supplementary Table S3—Allele Catalog Tool output *L1*). The remaining *G. soja* accessions also possess other mutations in *Glyma.19G120400* alongside the RI_indel (Supplementary Table S3—Allele Catalog Tool output *L1*). *G. max* accessions with the RI deletion (reference Williams 82, referred to as the H3 haplotype by Lyu et al. (2023)) exclusively displayed tan (32%), brown (66%), and light or dark brown (1% each) pod colors with no instance of black pods. This finding led us to believe that the RI_indel-only allele plays a pivotal role in the synthesis and accumulation of pigments perceived as the black color of pods. Based on this hypothesis, the presence of the RI_indel in *G. max* should lead to the black coloration of pods. After the reference allele and the RI_indel-only allele, the most frequent allele was a frameshift mutation K405fs found in 111 *G. max* accessions. The second and third most frequent alleles were two SNPs that lead to amino acid changes—R31C present in 71 varieties and G40D in 18 varieties. Additionally, two other frameshift mutations, L381fs and E402fs, were present in two accessions each.

To demonstrate MADis precision to specifically detect a causal gene for the example of *L1*, we performed the analysis for the nearest surrounding genes and a set of random genes using the same pod-color phenotypes. MADis scoring supported the selection of the causal gene based on the highest obtained score compared to the other tested genes (the scores for the nearest and random genes to validate the *L1* result are given in Supplementary Table S4). We demonstrated the precision of the tool to predict a causal gene and, therefore, confirmed that the tool can be used for the identification of uncloned genes with potentially multiple CMs.

### 3.3 Identification of an *L2* candidate gene

The *L2* locus controls brown or tan pod wall colors in conjunction with *l1* alleles (Palmer et al., 2016). We identified candidate genes for the *L2* locus by accuracy analysis with AccuCalc (Biová et al., 2023). Table 3 lists the highest accuracy variant positions in the candidate genes with their protein annotations. In the 1.5-Mbp genomic region that we analyzed as an extension of the *L2* locus, 650 modifying variant positions were identified as relevant based on their average accuracy higher than 50%. There were 187 tan pod-colored accessions and 418 brown pod-colored accessions that make up 56.8% of the known pod-color phenotypes in Soy1066. The reference cultivar Williams 82 (PI 518671) has tan pods and is *l1 l2*. Based on the highest average accuracy, we identified *Glyma.03G005700* as the candidate gene for *L2*, annotated as isopropylmalate synthase (IPMS; similar to the eucomic acid synthase GLYMA.19G120400). This gene has been identified as an *L2* candidate in a preprint (Li et al., 2023); it is a homolog to the cloned *L1 Glyma.19G120400* (Lyu et al., 2023).

**TABLE 3:** Top 20 candidate causative variant positions for pod-color *L2* candidate genes predicted by AccuCalc analysis (Biová et al., 2023). The table shows the AccuCalc analysis output for the *L2* locus, focused on *L2* TM ss715586818-associated genomic region of approximately ±1 Mbp, where only the top 20 highest average accuracy (Avr_acc)-modifying variant positions are displayed (NA, not assigned; Acc_Br, accuracy for brown pod-colored accessions; Acc_Tan, accuracy for tan pod-colored accessions; REF, reference genotype; ALT, alternate genotype; PFAM protein annotation (Punta et al., 2012); SoyCyc9, protein annotation) (Hawkins et al., 2021). A genomic region where multiple variant positions were identified in one gene is sub-sectioned.

Order	Position on Chr03	Distance to ss715586818	Avr_acc (%)	Acc_Br (%)	Acc_Tn (%)	REF	ALT	Gene	PFAM/SoyCyc9 annotation description	Effect
1	528,386	(8,443)	73.7	98.8	48.7	T	C	*Glyma.03G005700*	2-Isopropylmalate synthase	T3A
2	698,819	161,990	73.1	82.1	64.2	G	T	*Glyma.03G007500*	Myb-like DNA-binding domain	R27L
3	1,043,653	506,824	70.3	58.9	81.8	G	A	*Glyma.03G010800*	Bestrophin, RFP-TM, chloride channel	S446L
4	561,263	24,434	68.6	49.5	87.7	A	G	*Glyma.03G005900*	Co-chaperone GrpE family protein	S24P
5	926,521	389,692	68.6	48.3	88.8	G	T	*Glyma.03G009400*	Ca^2+^-dependent lipid-binding (CaLB domain) family protein	P19T
6	798,867	262,038	67.9	48.1	87.7	G	A	*Glyma.03G008200*	Major facilitator superfamily	S47F
7	798,850	262,021	67.9	48.1	87.7	G	T	*Glyma.03G008200*	Major facilitator superfamily	Q53K
8	571,097	34,268	67.5	62.2	72.7	T	C	*Glyma.03G006000*	Putative GTPase-activating protein for Arf	M483T
9	468,489	(68,340)	67.2	73.9	60.4	A	Insertion	*Glyma.03G005300*	MATE efflux family protein	Intron/splice donor variant
10	572,608	35,779	67.1	61.5	72.7	A	T	*Glyma.03G006000*	Putative GTPase-activating protein for Arf	T617S
11	842,426	305,597	67.1	66.8	67.4	G	A	*Glyma.03G008700*	NA	E92K
12	851,769	314,940	66.9	66.5	67.4	G	T	*Glyma.03G008800*	Protein kinase domain	S490R
13	852,396	315,567	66.9	66.5	67.4	T	C	*Glyma.03G008800*	Protein kinase domain	S374G
14	852,641	315,812	66.9	66.5	67.4	A	C	*Glyma.03G008800*	Protein kinase domain	Y322D
15	851,863	315,034	66.8	66.3	67.4	G	A	*Glyma.03G008800*	Protein kinase domain	S459F
16	851,744	314,915	66.8	66.3	67.4	C	T	*Glyma.03G008800*	Protein kinase domain	A499T
17	852,468	315,639	66.8	66.3	67.4	A	G	*Glyma.03G008800*	Protein kinase domain	Y350H
18	851,122	314,293	66.7	66.0	67.4	C	T	*Glyma.03G008800*	Protein kinase domain	A583T
19	402,682	134,147	65.8	71.8	59.9	G	A	*Glyma.03G004600*	Retinaldehyde-binding protein-related	A116T
20	403,903	132,926	65.7	71.5	59.9	A	G	*Glyma.03G004600*	Retinaldehyde-binding protein-related	K260R

### 3.4 Identification of CMs in *L2* alleles

**FIGURE 4:**
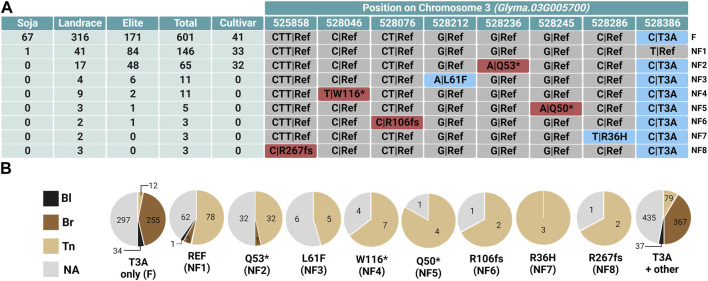
Allele distribution in *Glyma.03G005700* in Soy1066 and its correlation with different GRIN pod-colored phenotypes. **(A)** The table shows the selection of nine alleles identified by MADis, along with their positions in *Glyma.03G005700* and frequency counts of accessions carrying each allele. Positions with SNPs leading to amino acid change are highlighted in blue, while positions with frameshift mutations are indicated in red. Gray designates the reference (REF) allele, based on the Williams 82 reference genome. F indicates the presumptive functional allele; NF indicates the non-functional alleles (NF1–NF8). **(B)** Pie charts represent the distribution of pod-colored phenotypes for *G. max* accessions with specific alleles.

Based on the accuracy analysis, we observed the average accuracy of the T3A missense mutation in *Glyma.03G005700* as the highest among the candidate genes. The average accuracy is a mean of accuracies of WT and MUT accessions. For this particular variant position, we observed a nearly perfect correspondence between the brown pod-color phenotype and the Chr03:528, 386-C genotype (99%), whereas accuracy to the tan pod color was very low (49%). This led us to predict that there might be multiple CMs, as in the case of *L1 Glyma.19G120400*. First, we surveyed allelic variation present in the Soy1066 dataset using the Soybean Allele Catalog Tool. We detected 34 alleles of the *L2* candidate gene *Glyma.03G005700* in total (Supplementary Table S5—Allele Catalog output *L*2). Using MADis, we discovered eight independent putative CMs that corresponded to the complete loss of pigmentation in soybean pods, which results in the tan phenotype (Table 4). The full MADis result is given in Supplementary Table S6—L2 *Glyma.03G005700* MADis result. The Allele Catalog Tool output was simplified to show only positions identified by MADis (Figure 4A), and the pod-color phenotype distribution was analyzed for each allele (Figure 4B).

**TABLE 4:** Top five best-scored MADis outputs for *Glyma.03G005700*. The MADis results for the analysis of potential multiple CMs in *Glyma.03G005700* affecting the pod color trait. In the analysis, the 418 brown pod-colored accessions and 187 tan pod-colored accessions were used for MADis prediction, and the MADis result identified a perfect fit for more than 94% of analyzed accessions, implying the discovered multiple CMs in *Glyma.03G005700* as a strong candidate gene.

Order	Variant positions in *Glyma.03G005700*	Number of positions in the combination	Score	Brown (*n* = 418) explained	Tan (*n* = 187) explained	Explained phenotype (%)
1	525,858	8	533	411	158	94.1
	528,046					
	528,076					
	528,212					
	528,236					
	528,245					
	528,286					
	528,386					
2	525,858	9	531	410	160	94.2
	528,046					
	528,076					
	528,194					
	528,212					
	528,236					
	528,245					
	528,286					
	528,386					
3	525,858	9	531	410	158	93.9
	528,046					
	528,076					
	528,116					
	528,212					
	528,236					
	528,245					
	528,286					
	528,386					
4	525,858	9	531	410	158	93.9
	528,046					
	528,076					
	528,200					
	528,212					
	528,236					
	528,245					
	528,286					
	528,386					
5	525,858	7	529	411	156	93.72
	528,046					
	528,212					
	528,236					
	528,245					
	528,286					
	528,386					

The highest frequency count (601) was observed among accessions carrying the sole T3A allele (compared to the reference), which also includes the majority of *G. soja* accessions from Soy1066. The T3A variant position also co-occurs with other mutations. The T3A variant seems to be present in the ancestral functional *L2* allele that produces brown pods. The second highest frequency count is attributed to 167 accessions harboring the S172A variant alongside T3A. This allele is present in both *G. max* and *G. soja*; however, based on our MADis analysis for *L2*, this allele does not affect the protein function. The REF allele, based on the reference genome of Williams 82, is the third most frequent allele appearing in 146 accessions. This REF allele that contains a threonine at amino acid position three potentially encodes a non-functional protein, resulting in a loss of pigmentation and tan pods. Among the accessions with the REF allele, 78 accessions (93% of phenotyped accessions) were tan, but there were also five accessions with brown pods. There is a possibility that these accessions might have been wrongly phenotyped and are, in fact, tan. Although the majority of the accessions with the T3A variant alleles exhibit brown pods (367), there were 79 accessions with tan pods. Most of these with tan pods and T3A can be explained by the presence of other mutations in *Glyma.03G005700* that we identified by MADis. For the 12 accessions phenotyped with tan pods that solely featured T3A without other identified modifying variants, several possible explanations include unidentified genomic variation, an alternate mechanism that results in a non-functional protein, or the involvement of other regulatory genes somewhere in the pod color pigmentation pathway. The other seven *l2* alleles identified by MADis contained T3A plus one of the variants responsible for R36H, Q50*, Q53*, L61F, R106fs, W116*, or R267fs. These alleles were exclusively present in tan accessions, except for one accession carrying the Q53* allele with brown pods. This particular accession, labeled UN7_aka_Franklin (USA elite), might have been wrongly assigned PI548563 in the original resequenced dataset (Liu et al., 2020) and, therefore, might not decrease the reliability of our prediction. With an aim to strengthen our prediction, we determined the pod-color phenotype for the rare R267fs allele (NF8) in accession PI94159-3 and confirmed that it had tan pods (Supplementary Figure S7).

### 3.5 Natural and artificial selection of *l1* and *l2* multiple alleles

**FIGURE 5:**
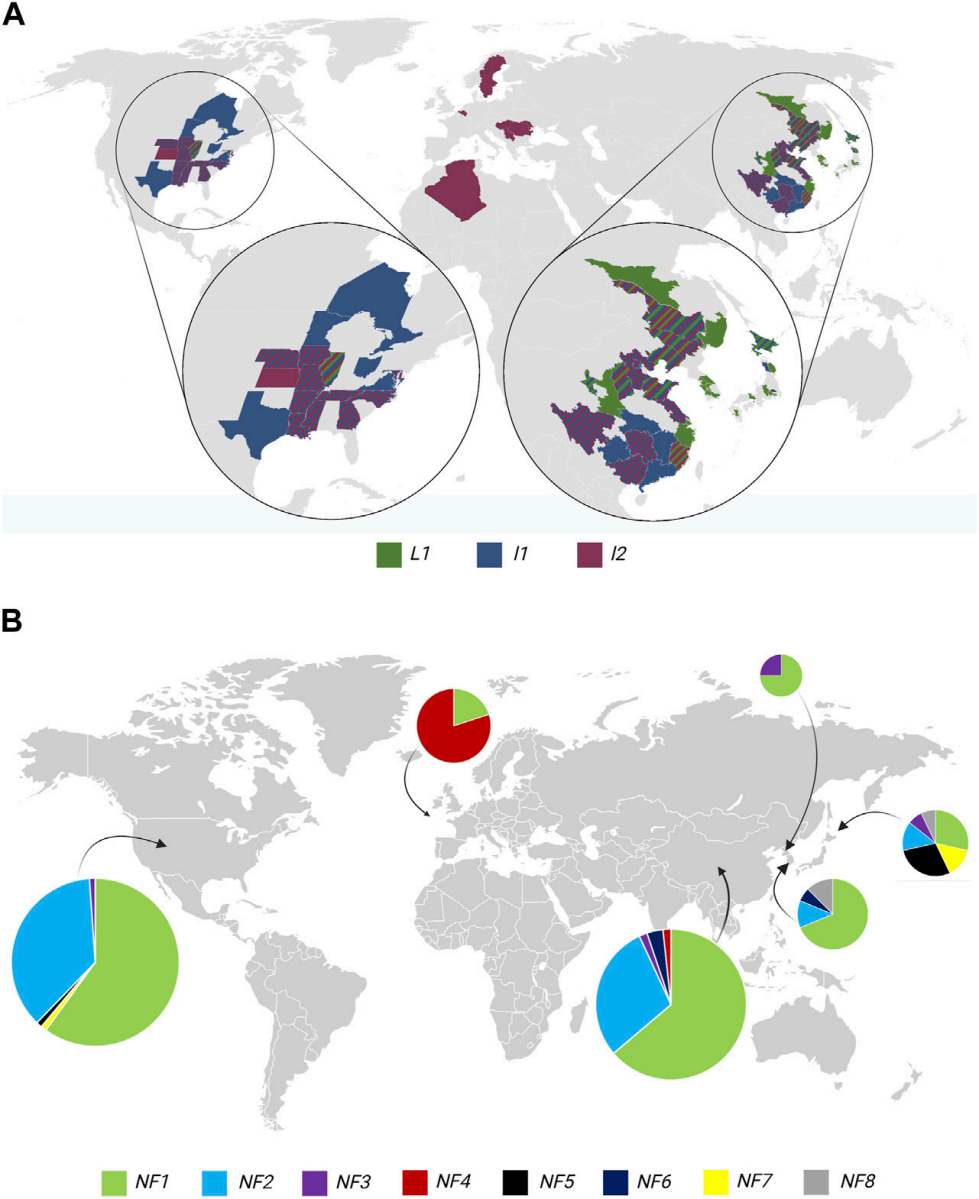
Geographical distribution of alleles responsible for pod color in soybean. **(A)** The global map displays the distribution of functional *L1*, non-functional *l1*, and non-functional *l2*. **(B)** Pie charts show the distribution of non-functional *l2* in six regions (United States, Europe, China, North Korea, South Korea, and Japan). The size of the pie charts represents the number of accessions with non-functional alleles in a specific region.

To assess natural and artificial variation of *L1* and *L2* and reveal the distribution of the alleles worldwide, we analyzed *l1* and *l2* alleles that contribute to brown and tan pod-color phenotypes using Allele Catalog analysis of the Soy1066 dataset (Supplementary Table S2—geographical information *L*2 and Supplementary Table S8—geographical information *L1*). We extracted accessions with available information about the geographical location of accessions (*n* = 430) and constructed a global map (Figure 5A). There were 161 accessions with the *L1* RI_indel ancestral allele that were previously described as an H1 haplotype (Lyu et al., 2023). The majority of accessions in Soy1066 with ancestral *L1* alleles were incorporated into GRIN with Chinese origin, while the majority of accessions with mutant alleles resulting in *l1* were present in accessions developed in the United States. We also observed that accessions with non-functional *l2* emerged all around the world. To thoroughly explore the origins of distinct non-functional *l2* alleles, we conducted a comprehensive analysis of their geographical distribution. We selected only accessions with available geographical information (*n* = 248) from the Soy1066 dataset and divided them into groups based on their region of origin and genotype. The distinct *l2* allelic distributions of each region were visually represented through pie charts embedded within a global map (Figure 5B). The NF1 allele, which corresponds to Williams 82, was found across all regions. Notably, the United States exhibited the highest frequency of NF1 (REF based on Williams 82) with 54 accessions, with the NF2 allele (Q53*) ranking as the second most prevalent. Interestingly, W116* was predominant in European soybeans (eight accessions), as well as in Algeria and China (one accession each). The NF5 (Q50*) allele was present in Japan (four accessions) and appeared also in the United States (one accession).

Although it is not yet understood what advantage lighter pod wall colors convey to cultivated soybeans, there are strong signals of selection for light pod wall colors resulting from the artificial selection of multiple independent non-functional combinations of *l1* and *l2* alleles (Figure 3; Figure 4). Soybean landraces in the Soy1066 dataset underwent selection for a spectrum of non-functional *l1* and *l2* alleles compared to the wild ancestor *G. soja* accessions, which almost universally displayed black pod walls and functional alleles of both *L1* and *L2*. The non-functional *l1* “not-black” alleles were nearly fixed in the elite accessions in the Soy1066 dataset, indicating that additional artificial selection at *L2* was possible for brown and tan pod walls. The most frequent allele for each of the improvement status categories in Soy1006 was the functional *L2* conditioning brown pod walls (when combined with *l1*). However, the frequency of tan pod wall non-functional *l2* alleles increased from the landrace (20%) to the elite (45%) improvement category in the Soy1066 accessions (Figure 4).

## 4 Discussion

Natural and artificial selection can result in multiple independent alleles as CMs for phenotypes due to convergent evolution. The origin context and frequency of those alleles can complicate analysis methods to identify genes that underlie phenotypes. Biparental genetic mapping is a powerful approach to connect phenotypes to either one of the two parental alleles of candidate genes, but the analysis is limited to only those contrasting parental alleles. When GWAS is used on diverse accession panels, rare alleles can be overlooked or masked by the major frequency allele. The slow progress in identifying CMs after GWAS and complexities with our own post-GWAS analysis studies led us to develop the MADis analysis tool to investigate whether or not multiple alleles of an identified candidate gene are in play. MADis is an extension of the accuracy concept that we implemented to the Synthetic Phenotype to Causative Mutation (SP2CM) strategy (Škrabišová et al., 2022) for more efficient GWAS-driven discoveries. Using accuracy, a variant position is considered a possible CM based on its highest correspondence with a phenotype. Therefore, low-average accuracy variant positions are omitted from the candidates, regardless of their perfect correspondence with the WT category (ancestral phenotype). In our previous work (Škrabišová et al., 2022), we demonstrated that if one of the multiple alleles is prevalent in a dataset with a high frequency, its variant position can be identified as causative, as in the case of multiple alleles in the stem termination type *Dt1* gene (Liu et al., 2010; Tian et al., 2010).

Seven polymorphisms were found in the *L1* pod color-underlying gene, but only three haplotypes were proposed to play a role in the loss of fully black pigmentation (Lyu et al., 2023). Together with the Williams 82 reference haplotype H3, the three alternate haplotypes were the most frequent ones among the 20 alleles. Here, we showed that thorough analysis of the distribution of the allele in improvement status categories is potent in predicting additional CMs in rare alleles, such as in the case of previously identified G40D. This polymorphism is present in the MADis highest score selection of the six independent multiple CM positions (Table 2, Supplementary Table S3). Thus, G40D is the fourth most frequent allele of *l1*. On the contrary, the N418D and T196S polymorphisms (Supplementary Table S3) are not causative since these are present in the functional version of IPMS in some of the *G. soja* accessions. The V276A polymorphism is part of the non-functional RI_indel-containing *l1* allele and, therefore, cannot be evaluated for impact on the protein function. Our analysis showed that MADis can detect very rare polymorphisms that fall under a low minor allele frequency (MAF) limit (MAF<3%). Although MADis is limited by the binomial distribution of phenotypes, this is in accordance with the bi-allelic nature of the vast majority of genetic variants (Sachidanandam et al., 2001). Here, it has to be noted that MADis prediction significance is determined by the number of samples in the analysis and, therefore, should be performed on large diverse datasets to maximize the power of prediction.

We identified an *L2* candidate gene based on the accuracy and multiple CMs based on MADis scoring. As shown in Table 3, *Glyma.03G005700* has been detected as the most probable candidate among other highly associated genes. Although no identity was observed between this gene and the previously proposed candidates (Song et al., 2004; Lemay et al., 2023), it is annotated as an isopropylmalate synthase, a homolog to *L1*, the eucomic acid synthase (Lyu et al., 2023). It recently came to our attention that the same gene that we identified as *L2* was also identified for *L2* in a non-peer-reviewed preprint (Li et al., 2023). This work is in accordance with our identification and multiple CM prediction and might be a valuable confirmation of our analyses.

To assess the natural and artificial selection of the three main pod-colored phenotypes, we examined the distribution of *L1/l1/l2* phenotypes in the context of the world soybean population (Figures 5A, B) based on our diversity panel Soy1066 (Chan et al., 2023). Although we are aware of the limitations of Soy1066 in terms of information about the countries of origin, the distribution of functional *L1* black pod-colored genotypes was centered more to the regions of soybean origin (Figure 5B; China, Korea, and Japan), whereas *l1* and *l2* genotypes were selected in geographical regions where soybeans were introduced, domesticated, and adopted. The geographical distribution of *l1* and *l2* genotypes is in accordance with the previously observed correlation of *l1* genotypes with lower pod shattering (Lyu et al., 2023) and, thus, suggests artificial selection for this trait.

To the best of our knowledge, there is currently a dearth of available strategies that could be deployed for the identification of multiple CMs in candidate genes even though this is a frequent problem in GWAS discoveries. In human genetics, a variety of association models was recently specified to cover different genetic architectures with the aim to test allelic series for the identification of rare variants (McCaw et al., 2023). The predicted cross value was proposed to solve the challenge of introgressing multiple alleles in crop genetic improvement (Han et al., 2017). A multi-allele haplotype prediction was used on a wheat training population and suggested for multiple allele prediction regarding the linkage disequilibrium for self-fertilizing crops (Sallam et al., 2020). The MADis analysis scores combinations of variant positions, regardless of the linkage disequilibrium in known loci and, therefore, enables discoveries unlimited by the population structure, directly in candidate genes. Soybean MADis is gene-oriented; however, the MADis Python package is publicly available and can be used for analysis of variant positions beyond genic regions. In our future work, we will test the MADis algorithm for its potential utilization in predicting variation in promoter regions.

## 5 Conclusion

In this work, we solved the GWAS-limiting factor caused by the existence of parallel CMs in candidate genes that arose during natural and artificial selection. We developed, tested, and validated the MADis tool for the successful identification of multiple CMs for soybean. The MADis analysis platform is publicly available for other species to aid in the discovery of genes under selection for accelerated and, thus, improved breeding.

## Data Availability

Publicly available datasets were analyzed in this study. The Soy1066 dataset can be freely downloaded at https://soykb.org/public_data.php.

## Supplementary material

SUPPLEMENTARY TABLE S1 MADis result for L1 Glyma.19G120400.

SUPPLEMENTARY TABLE S2 List of the Soy1066 accessions with the country of origin and L1 allele status.

SUPPLEMENTARY TABLE S3 Soybean Allele Catalog output for L1 Glyma.19G120400.

SUPPLEMENTARY TABLE S4 Validation of MADis.

SUPPLEMENTARY TABLE S5 Soybean Allele Catalog output for L2 Glyma.03G005700.

SUPPLEMENTARY TABLE S6 MADis result for L2 Glyma.03G005700.

SUPPLEMENTARY TABLE S7 Comparison of the pod color phenotypes of Clark and PI 94159-3.

SUPPLEMENTARY TABLE S8 List of the Soy1066 accessions with the country of origin and L2 allele status.
