# Supplementary material for: Natural and artificial selection of multiple alleles revealed through genomic analyses
Source: Front Genet. 2024 Jan 8;14:1320652. doi: 10.3389/fgene.2023.1320652 (PMC10801239; doi:10.3389/fgene.2023.1320652)
Supplement: Supplementary file 3 [file Table1.DOCX]

***Supplementary Material***

Natural and Artificial Selection of Multiple Alleles Revealed Through Genomic Analyses

**Jana Biová, Ivana Kaňovská, Yen On Chan, Manish Sridhar Immadi, Trupti Joshi, Kristin Bilyeu, Mária Škrabišová**

*** Correspondence:**Kristin Bilyeu: kristin.bilyeu@usda.gov; Mária Škrabišová: maria.skrabisova@upol.cz

# Supplementary Data

**Phenotyping for pod color**

In 2023, the PI 94159-3 was obtained from the USDA Soybean Germplasm Collection (GRIN, Urbana, IL) and a single plant was started from seed, transplanted, and grown to maturity in a field plot at the South Farm Research Center near Columbia, Missouri, USA. Pod wall colors were compared with other *G. max* accessions grown in the same environment.

# Supplementary Figures and Tables

1. **Supplementary Figures**

**
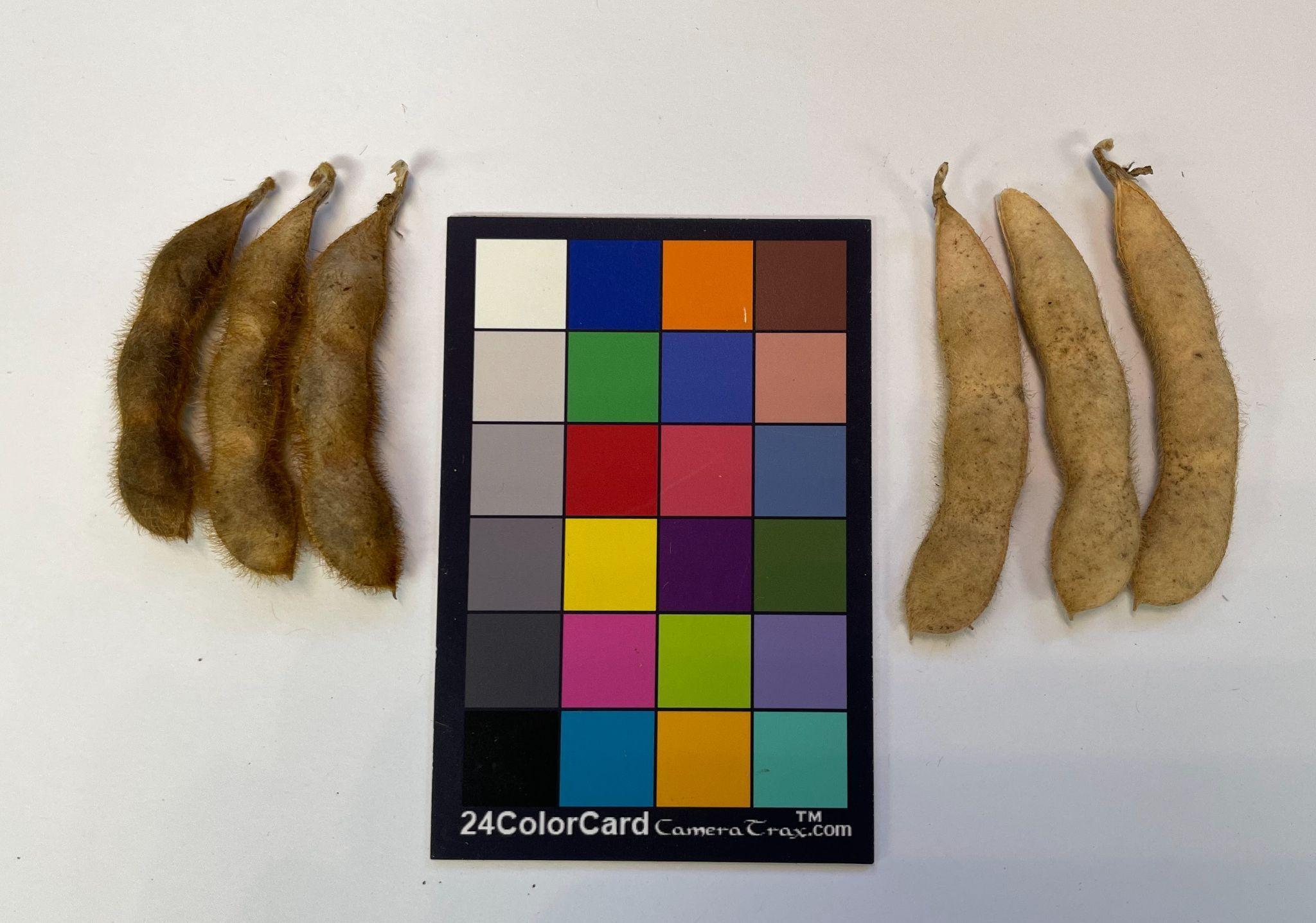
**

**Supplementary Figure 1.** Comparison of the pod color phenotypes of Clark and PI 94159-3. Clark (left) has brown pod color and tawny pubescence which is consistent with the GRIN database. In contrast, PI 94159-3 (right), a genotype with no pod color phenotype data in GRIN, displays a tan pod color and grey pubescence. Our preliminary analyses of recently resequenced soybean accessions with *L2* NF8 support our finding that R267fs might be a non-functional version of IPMS since there are an additional nine accessions with this allele that are all tan pod-colored.
